# Gender differences in the subjective wellbeing of the older adults and the determinant factors: a case study of Nanjing

**DOI:** 10.3389/fpubh.2024.1447777

**Published:** 2024-08-30

**Authors:** Jianxi Feng, Min Zheng

**Affiliations:** Department of Urban and Regional Planning, School of Architecture and Urban Planning, Nanjing University, Nanjing, China

**Keywords:** subjective wellbeing, older adults, gender differences, built environment, household structure

## Abstract

**Objective:**

This paper aims to examine the gendered differences in the subjective wellbeing of older adults and underlying determinant factors which contribute to these differences in China where the unique social and cultural systems, the consequent concept of filial piety and the perceptions towards different living arrangements in later life provide an excellent laboratory for studying the topic.

**Methods:**

Hierarchical linear models are employed to analyze the impacts of household structure and built environment on the subjective wellbeing of older adults based on a survey conducted in Nanjing in 2021.

**Results:**

There are significant gender differences in the subjective wellbeing of older adults, with older women reporting higher levels of subjective wellbeing (4.95 vs.4.69). Gender differences also exist in how the built environment affects the subjective wellbeing of older adults, with a greater impact on older adult women (33.68% vs. 28.50%). Household structure impacts the subjective wellbeing of older adults through the division of housework and the company of family members.

**Conclusion:**

There are three major mechanisms through which gender affects the subjective wellbeing of older adults, including structural mechanisms, socio-cultural mechanisms, and physiological mechanisms. Targeted environmental interventions and urban planning policies are recommended to promote the subjective wellbeing of older adults.

## 1 Introduction

Subjective wellbeing (SWB) is an important indicator for measuring the mental health of older adults (1, 2). It is also a critical criterion for assessing “healthy aging”. In the era that the whole world is experiencing fast aging, enhancing the SWB of older adults is crucial for improving their mental health and constructing an age-friendly society. The rapid increase in both the proportion and absolute number of older adults in China will have a series of impacts on the entire society (3, 4). The huge gender differences in life expectancy between men and women have resulted in an imbalanced population proportion of older men and women, suggesting that public policies aimed at enhancing the wellbeing and health status of older adults need to account more for gender differences (5, 6).

In recent decades, gender inequality in SWB has been a hot topic of research. There are also many studies that have examined the factors influencing the SWB of older adults (7, 8). Yet, studies focusing on gender differences in the SWB of older adults and the determinant factors are relatively scarce. There are only few exceptions that scholars have explored the impacts of leisure activities participation (9, 10), social involvement (9, 11), intergenerational support (12), and learning behaviors (13) of older adults on the gender differences in SWB. Other factors, including the built environment and household structure, are rarely examined. Previous research has indicated that built environment and household structure are significant factors for the SWB of older adults (14–16), and it seems that they influence the wellbeing of the old men and old women differently. However, to the best of our knowledge, there are still no studies that have comprehensively examined how built environment and household structure are associated with SWB and particularly how these associations vary with gender.

Due to substantial differences in social systems, cultural norms, gender roles and the built environments (17–20), gender differences in SWB among older adults and the determinants might differ across countries (21–23). China's unique social and cultural systems, the consequent concept of filial piety and the perceptions toward different patterns of older adult care and living arrangements in later life as well as the distinctive built environments have resulted in different understandings and preferences regarding SWB from their counterparts in other countries. These differences cast doubt on the applicability of empirical research results and policy recommendations based on cases abroad. In other words, China's distinctive context provides an excellent laboratory for exploring gender differences in the SWB of older adults and can offer a unique perspective to examine the determinant factors of SWB. Therefore, research on gender differences in SWB among older adults in Chinese society and its determinants are important for deepening academic understanding of SWB, as well as for formulating targeted intervention policies to enhance the wellbeing of older adults.

To bridge the above academic gap, this paper attempts to answer the following questions: Are there gender differences in the SWB among older adults people in China? How do household structure and the built environment affect the gendered SWB perception among older adults? To answer these questions, this paper employs a hierarchical linear model to analyze the gender differences in the impacts of built environment and household structure on the subjective wellbeing of older adults based on a survey of the quality of life of older adults in Nanjing.

The reminder of the paper is structured as follows: Section 2 reviews the relevant literature on gender differences in SWB among older adults. Section 3 starts with an overview of the data, measures of the variables and the methods used. In Section 4, we turn our attention to an analysis of our dataset and a number of regression models. Section 5 delves into the discussion of the analysis results, shedding light on the mechanisms of the gender differences in SWB. Finally, conclusions are drawn in Section 6.

## 2 Literature review

The differences between SWB of men and women has received increasing attention in the academic communities of gerontology, psychology, and health geography (17, 18, 24). Nonetheless, these studies have not reached consistent results, with some studies found that the level of SWB of women is higher than that of men and others found the reverse (25–27). There are also studies that find no significant gender differences in SWB (28, 29). Moreover, gender differences in SWB may vary with age, i.e., the increase in age may have different effects on the SWB between men and women (30).

Relatively few studies have been dedicated to the gender differences in SWB of the old people. According to existing studies, there are interactions between gender, age and wellbeing (30, 31), suggesting a complex picture of gender differences in SWB of older adults. A metal-analysis by Pinquart and Sorensen (32) concluded that the relationship between gender and wellbeing tends to reverse as age increases, and older women reports markedly lower SWB than men. However, those studies focusing on this issue have mostly stemmed from western contexts. Less attention has been paid to the situation of China, resulting in incomplete understandings of the gender differences in the wellbeing of the older adults.

With respect to the factors that contribute to the gendered differences in SWB, there are even fewer studies focusing on this topic with only few exceptions. Li et al. (11) and Zhang et al. (10) found that social activities were more beneficial to SWB for males than for females. Regarding intergenerational support, the financial support offered by the offspring plays a much more crucial role in SWB for older women than for older men, while caregiving support matters more to men than to women (12). With respect to learning behaviors, Shi et al. (13) concluded that the positive impact of learning behaviors on subjective wellbeing of older women was more pronounced compared to older men. Due to declines in physical function and cognitive capacity (33), older adults' daily activity spaces shrink, making them highly reliant on the built environment at the community level (34, 35). In other words, the built environment at the community level tend to play a much more vital part in improving older adults SWB (16, 36) than other population sections. Moreover, women are reported to be more sensitive to the local environment for physiological differences (37). Regarding household structure, previous studies have shown that it can affect the wellbeing of older adults through domestic roles (9, 38) and the consequent division of household responsibilities (21, 39), emotional bonds (40, 41) and social appraisal of the eldercare model (42, 43), yet whether it impacts the old men and old women in the same way and to the same extent remains unexplored.

At present, aging-in-place remains the mainstream eldercare model in China, meaning that both the built environment at the community level and household structure are important factors affecting the SWB of older adults. It is therefore necessary to explicitly investigate how and why the effects of these factors on senior's happiness vary with gender. Base on previous studies, the gender-differentiated effect of built environment and household structure on senior's subjective wellbeing might be attributed not only to physiological differences between men and women, but also to the gender disparities, socio-cultural norms, the division of household labor and thus the differing needs of living environments, etc. For example, in China, women generally take on much more household responsibilities than men (44, 45), and this gendered divisions of household labor continue into late life (39, 46). The household responsibilities not only directly affect older adults wellbeing, but also make them perceive the surrounding built environment differently (47). However, only a few studies have examined the gender differences in older adults wellbeing and its determinants, the underlying mechanisms still remains to be fully explored.

Therefore, in this study we will address the above research gap by exploring the gender differences in the SWB among the old males and females in China and investigating the gender-differentiated effects of built environment and household structure. Our study seeks to provide a complete picture of the gender differences in SWB and its determinants in China while also advance the theoretical discussions in happiness studies.

## 3 Materials and methods

### 3.1 Study area and data

Mega-cities in China are the areas where the senior population concentrated (48), and therefore are the ideal places for studying academic issues related to aging society. Nanjing, located in the eastern coast region of China, is not only the capital city of Jiangsu Province and an important central city in Eastern China, but also the sub-center of the Yangtze River Delta Mega-City Regions. In 2015, the population of Nanjing was ~8.23 million. The people who are aged 65 and above accounted for 10.69% of the total population, indicating that Nanjing has entered an aging society (49). By the end of 2021, the population of Nanjing had reached about 9.42 million, with the amount of older adults who are 65 years and above rising to a percentage of 14.49%. According to the national statistics, the percentage of older adults people and the speed of aging in Nanjing is quite typical among the Mega-cities in developed area in China and therefore be selected as the study area.

We analyzed the connections between the built environment and the SWB of older adults based on a survey called “The quality of life of older adults in Nanjing”, which was conducted between September and November 2021. Actually, we had conducted almost the same survey in the same selected communities in 2015. The newly conducted survey is the continuation of that research. The survey was also funded by a national grant. It was designed to investigate how the built environment influences the quality of life of older adults. The questionnaire includes the socio-economic attributes of older adults (including age, income, marital status and household structure, etc.), involvements of household responsibilities, their daily travel-activity information and subjective wellbeing.

**Figure 1:**
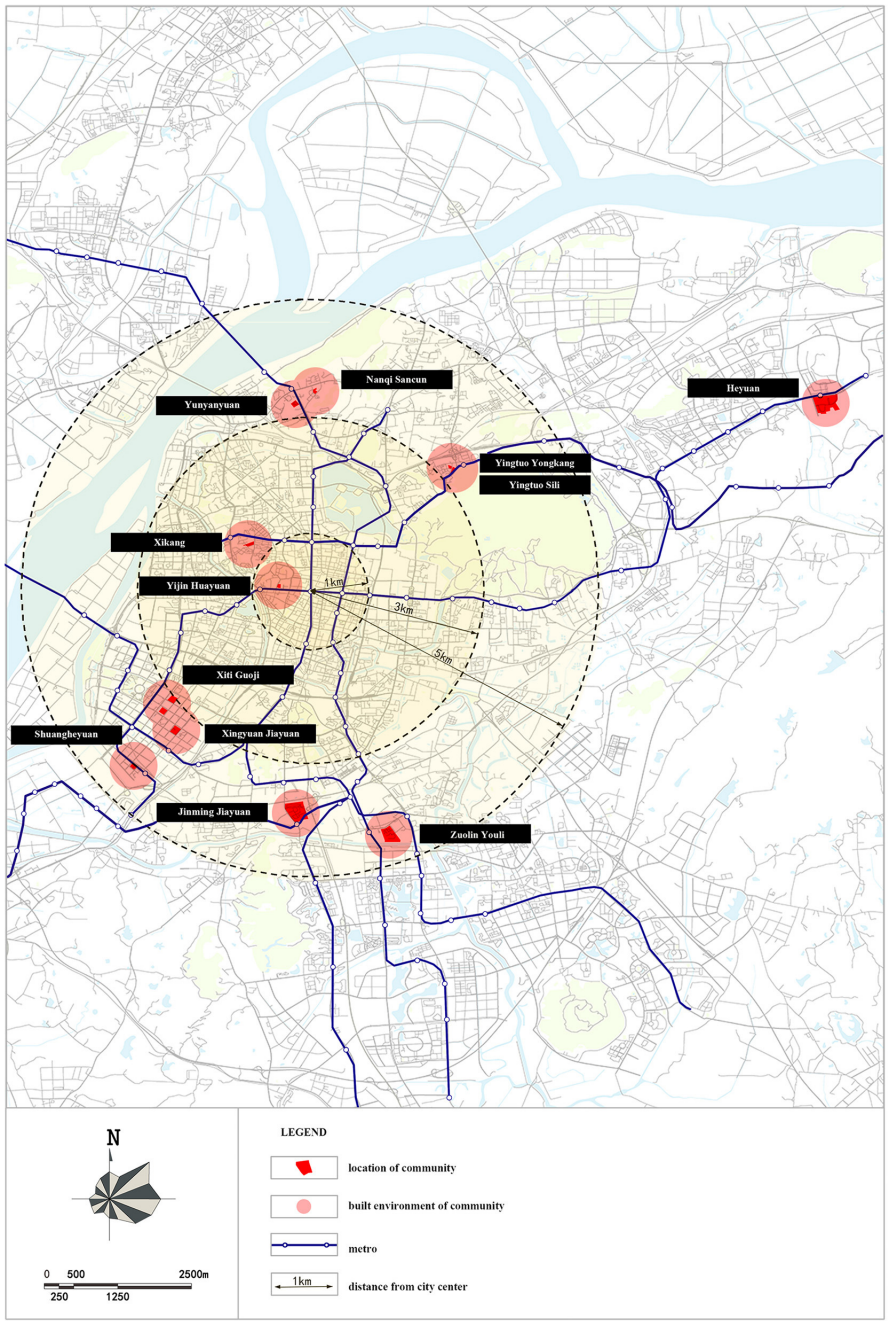
The location of 12 typical communities.

We utilized a stratified sampling approach for the survey execution. Initially, we examined various communities across the entire metropolitan region of Nanjing, noting the diverse characteristics of their built environment. Informed by this assessment, we identified and chose representative communities. Subsequently, we performed random sampling of the senior population within these selected areas. Employing such informed stratification reduced the variance inside each strata compared to the total population variance, therefore producing better strata efficiency than systematic sampling or random sampling methods (50, 51). For our study, a total of 12 communities were chosen to typify the diversity in built environment, considering both location and the internal and external conditions of the communities (Figure 1; Table 1). In each community, we drew a random sample of about 50 senior respondents that we either encountered in their communities or surveyed at their homes. In total, we collected data from 640 individuals. The final, valid sample includes 617 respondents, and the percent of the valid sample is 96%. Before examining the interrelations among household structure, built environment, and the subjective wellbeing of older adults, we first tested the validity and reliability of the data with SPSS 25. The *P*-value of the Bartlett's test was < 0.05, the Kaiser-Meyer-Olkin (KMO) score was 0.786, and Cronbach's alpha value was 0.755, suggesting that the survey questionnaire has good validity and reliability.

**Table 1:** Profile of the samples.

**Attributes**	**Categories**	**Proportion (%)**	**Mean/SD**
**Socio-economic attributes**			
Gender	Male (=ref.)	39.61	
	Female	60.39	
Age	60–65 (=ref.)	33.06	
	66–70	34.21	
	Older than 71	32.73	
Education attainment	Illiteracy (=ref.)	9.17	
	Primary school	13.09	
	Middle school	33.72	
	High school or technical secondary school	25.37	
	Junior college, undergraduate and above	18.65	
Income (RMB per month)	0 (=ref.)	3.60	
	< 1,000	7.20	
	1,001–3,000	44.19	
	3,001–5,000	27.17	
	More than 5,001	17.84	
Hukou status	Local people	67.43	
	Non-local people (=ref.)	32.57	
Communist party membership	Yes	22.09	
	No (=ref.)	77.91	
Household structures	Single	8.02	
	Couple	32.08	
	Living with daughter	9.00	
	Living with daughter-in-law (=ref.)	6.71	
	Living with daughter and grandchildren	9.82	
	Living with daughter-in-law and grandchildren	21.11	
	Others	13.26	
**Built environments**			
Population density	Population per unit area of community (10,000/km^2^)		2.04/1.20
Land use mix	The extent to which facilities are evenly distributed within the 500-meter buffer zone around the community		0.55/0.11
Accessibility of open space	Straight-line distance from the community to the nearest open space (m)		1,404.26/593.28
Accessibility of kindergarten	Number of kindergartens within 1,000 meters of the community		7.36/5.09
Accessibility of public transport	Number of metro stations within 1,000 meters of the community		1.09/0.76

### 3.2 Research framework and methods

**Figure 2:**
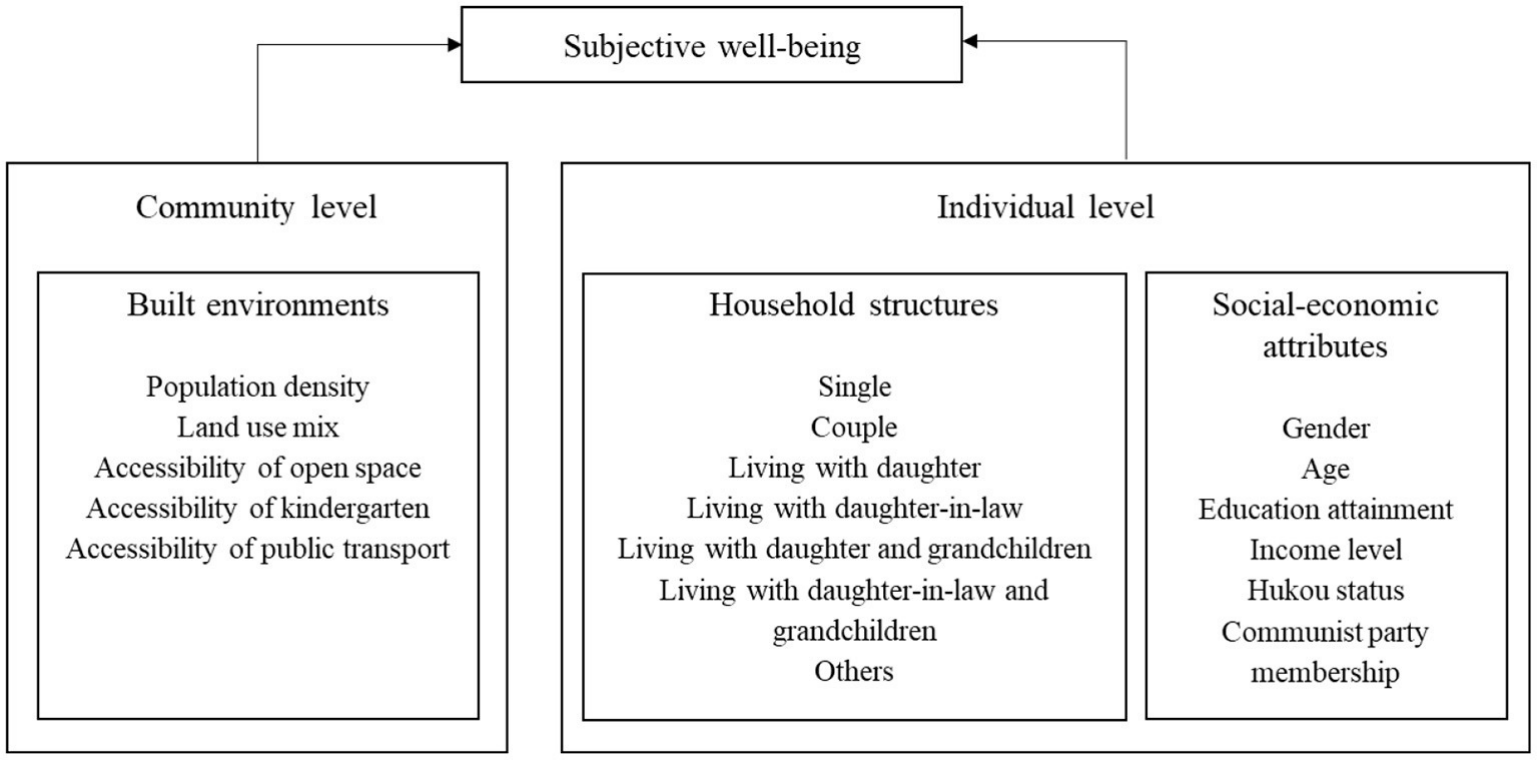
The research framework of HLM.

As shown in Figure 2, this paper will analyze the gender differences in SWB among older adults and to detect the different impacts of built environment at the community level and household structure at the individual level. To realize this research aim, Hierarchical Linear Model (HLM) is employed and other socio-economic characteristics of older adults is controlled including age, education attainment and income level. Additionally, two factors with particular Chinese characteristics (hukou status and communist party membership) are selected in the article.

HLM is useful for understanding the relationships in hierarchical data structures. Data are collected on random samples of older adults nested within each community. In this application, it might be appropriate to adjust for covariates at both the individual-level (age, gender, education attainment, income level, hukou status, communist party membership and household structure) and at the community-level (built environments). The model begins with a fixed slope, random intercepts model, expressed as:


(1) $$\begin{array}{r} First\;level:y_{ij}=\beta_{0j}+\beta_{1j}gender_{ij}+\;\beta_{2j}household_{ij}\\ +{\;\beta }_{kj}individual_{ij}+e_{ij} \end{array}$$

(2) $$\begin{array}{l} Second\;level:\beta_{0j}=\gamma_{00}+\gamma_{01}environment_{j}+\mu_{0j} \end{array}$$

Here (Equation 1), *y*_*ij*_ represents the subjective wellbeing of older adults, that is, the subjective wellbeing of the *i* individual in the *j* community. The first level is the individual-level equation; β_0*j*_ is the community-level intercept, representing the average subjective wellbeing of older adults at the community level; *gender*_*ij*_ denotes the gender of older adults, *household*_*ij*_ indicates their household structure, and *individual*_*ij*_ represents other individual demographic and socio-economic attributes, with *e*_*ij*_ being the individual-level random error. The second level (Equation 2) is the community-level equation; γ_00_ is the mean of β_0*j*_, that is, the overall average score of older adults subjective wellbeing, *environment*_*j*_ is the built environment indicator at the community level, and μ_0*j*_ is the community-level random error.

The implicit assumption of the above model is that the effects of community-level built environment on the relationship between individual-level variables and subjective wellbeing is constant. However, the impact of gender on the subjective wellbeing of older adults may vary with the built environments. Therefore, we construct a random slope, random intercepts model to analyze how built environments affect the relationship between gender and subjective wellbeing of older adults (Equations 3, 4). This involves interacting gender with the built environment variables, keeping the second level unchanged, while the first level model is expressed as:


(3) $$\begin{array}{r} First\;level:y_{ij}=\beta_{0j}+\beta_{1j}gender_{ij}+\;\beta_{2j}household_{ij}\\ +\;\beta_{kj}individual_{ij}+e_{ij} \end{array}$$

(4) $$\begin{array}{l} Second\;level:\beta_{1j}=r_{10}+r_{11}community_{j}+\mu_{1j} \end{array}$$

### 3.3 Measures

In this study, SWB is measured using the widely utilized and readily available Satisfaction With Life Scale (SWLS) (52). The five SWLS statements are: “In most ways, my life is close to my ideal,” “I am satisfied with my life,” “So far, I have achieved the important things I want in life,” “The conditions of my life are excellent” and “If I could live my life over again, I would change almost nothing” (Table 2). The questionnaire records respondents' answers using a Five Point Likert Scale, where 1–5 points represent “strongly disagree,” “disagree,” “neutral,” “agree,” and “strongly agree,” respectively. Principal Component Analysis (PCA) is then conducted to obtain the dependent variable of subjective wellbeing (continuous variable) used in the final model. The Kaiser-Meyer-Olkin (KMO) value is 0.908, and the *P*-value of Bartlett's test is < 0.01, indicating that PCA is an appropriate method.

**Table 2:** Measurement of subjective wellbeing indicators.

	**Questions**	**Mean/standard deviation**	**Minimum**	**Maximum**
SWLS	In most ways, my life is close to my ideal	2.66/0.63	1	5
	I am satisfied with my life	2.71/0.57	1	5
	The conditions of my life are excellent	2.56/0.73	1	5
	So far, I have achieved the important things I want in life	2.46/0.81	1	5
	If I could live my life over again, I would change almost nothing	2.68/0.61	1	5

Based on the “5Ds” of the built environment (53) and considering data availability, this paper developed five built environment variables: population density, land use mix, accessibility of open space, accessibility of kindergarten and accessibility of public transport. Population density is calculated as the ratio of population and area of the community in Nanjing. Land use mix simultaneously accounts for the variety and prevalence of different functions in the area. Following Ewing and Cervero (53), we calculated an entropy index:


$$\begin{array}{l} S=-\underset{j}{\sum }\frac{\left[P_{jk}\times \ln\left(P_{jk}\right)\right]}{\ln\left(J\right)} \end{array}$$

In this equation, *S* refers to land use mix (entropy); *j* is the type of land use (*j* = *1, 2, …, J*); *k* is the community (*k* = *1, 2, …, K*); *P*_*jk*_ is the proportion of land use *j* within the community. The entropy ranges from 0 (homogeneity-only one type of land use) to 1 (heterogeneity-shares of uses evenly distributed over all land use categories). We include six land use types with highest relevance for residents' daily activities: residential, commercial, public, industrial, offices and research sites, and parks and recreational use. Accessibility of open space is calculated by the straight-line distance to the nearest green square. Accessibility of kindergarten/public transport is calculated by the number of kindergartens/metro stations within a straight-line distance of 1 kilometer from the center of the community, respectively.

Household structure is an important factor affecting the subjective wellbeing of older adults. In contrast to the west, Chinese society places a high priority on aging-in-place, and there are still a substantial portion of Chinese seniors living together with their married children. Sons bear main responsibility for taking care of their parents, while daughters need to care for her husband's parents as a daughter-in-law after marriage. Existing research also found that there are great differences between living with daughter and living with daughter-in-law on the subjective wellbeing of older adults (54). In the light of the above reasons, this paper categorizes household structure into seven types namely “Living alone,” “Living with spouse,” “Living with daughter-in-law,” “Living with daughter,” “Living with daughter-in-law and grandchildren,” “Living with daughter and grandchildren,” and “Others.” The analytical results of the paper show that different types of household structures tend to have significant different consequences in shaping the level of senior's SWB, indicating the validity of our classification.

## 4 Results

### 4.1 Gender differences in SWB of older adults

As shown in Table 3, there are differences in SWB between older adult men and women, with women having a higher average level than men (4.95 vs. 4.69). Mann-Whitney *U*-test shows that the difference in subjective wellbeing between older adult men and women is significant (*P* < 0.01). Additionally, the larger standard deviation suggests that the distribution of SWB score among older adult men is more polarized, whereas the distribution among older adult women is relatively steady. There might be several reasons accounting for the gendered discrepancies in SWB. Firstly, women and men may have different expectations regarding aging and contentment. This could shape their self-reported feelings of wellbeing, with women perhaps having more adaptable or realistic expectations compared to men. Secondly, in China women are often seen as being more resilient and better at maintaining social networks. They are more likely to express their feelings and seek help when facing emotional difficulties, which can contribute to emotional support and satisfaction in later life. Men, on the other hand, may have traditionally derived much of their identity and satisfaction from their professional roles, and retirement could disrupt this source of fulfillment. The polarization among men could reflect a reluctance to seek help or discuss emotional issues, resulting in more extreme levels of reported SWB or dissatisfaction. Thirdly, family and living arrangements might also contribute to the discrepancies: older adult women may benefit more from household structures that provide emotional and practical support; men might experience more loneliness if widowed, as they may be less likely to maintain extensive social networks outside of marriage. In other words, these gender discrepancies in SWB among older adults likely stem from a complex interplay of social-cultural, physiological-psychological, and economic factors. Therefore, in next subsection, we adopted multilevel regression models to detect the underlying factors.

**Table 3:** Gender differences in SWB.

**Subjective wellbeing**	**Mean**	**Standard deviation**
Male	4.69^***^	1.03
Female	4.95^***^	0.93

^***^p < 0.01.

### 4.2 Determinant factors of the gendered differences in SWB of older adults

This paper adopted hierarchical linear regression models to analyze the gender differences in subjective wellbeing and their influencing factors using software of Stata 15.0 and SPSS 25. Before modeling, multicollinearity diagnostics is performed, yielding a Variance Inflation Factor (VIF) < 5, indicating no multicollinearity among the independent variables. To test the applicability of the multilevel model, an empty model is constructed to examine whether there are intra-class differences among the samples. Results show that the inter-class variance for the empty model is 0.3029, and the intra-class correlation coefficient (ICC) value is 0.3172, meaning that community differences account for 31.72% of the total variance in senior's subjective wellbeing and the explanatory power of the multilevel model is significantly higher than that of single-level models (55). Then the hierarchical linear regression models are conducted and the results are shown in Table 4. It can be observed that the overall effect of gender on senior's SWB is significantly validated (Model 2 and Model 3), with older adult women having notably higher levels of subjective wellbeing than men, indicating that even after controlled for other variables, gender differences in SWB of older adults still exist.

**Table 4:** Hierarchical linear model of subjective wellbeing of older adults.

**Independent variables**	**Empty model**		**Model 1**		**Model 2**		**Model 3**	
	**Coefficient**	**S.E**.	**Coefficient**	**S.E**.	**Coefficient**	**S.E**.	**Coefficient**	**S.E**.
**Household structures (living with daughter-in-law** = **ref.)**								
Single					−0.3055^*^	0.1729	−0.2989^*^	0.1727
Couple					−0.2080	0.1408	−0.2236	0.1404
Living with daughter					−0.2129	0.1652	−0.2278	0.1648
Living with daughter and grandchildren					−0.3279^**^	0.1654	−0.2676^*^	0.1447
Living with daughter-in-law and grandchildren					−0.2826^*^	0.1453	−0.3200^*^	0.1649
Others					−0.1313	0.1559	−0.1323	0.1553
**Social-economic attributes**								
Gender (Male = ref.)					0.2356^***^	0.0748	0.2355^***^	0.0746
**Age (60–65** = **ref.)**								
66–70					0.1497^*^	0.0872	0.1361	0.0874
Older than 71					0.0292	0.0876	0.0277	0.0874
**Education attainment (Illiteracy** = **ref.)**								
Primary school					0.2392^*^	0.1431	0.2218	0.1429
Middle school					0.2708^**^	0.1285	0.2630^**^	0.1281
High school or technical secondary school					0.2066	0.1388	0.2151	0.1384
Junior college, undergraduate and above					0.2031	0.1578	0.1933	0.1575
**Income level (0** = **ref.)**								
< 1,000					−0.0982	0.2099	−0.1194	0.2101
1,001–3,000					−0.1213	0.1843	−0.1288	0.1837
3,001–5,000					−0.0983	0.1950	−0.0804	0.1944
More than 5,001					−0.0398	0.2076	−0.0507	0.2074
Hukou status (non-local people = ref.)					0.0795	0.0847	0.0801	0.0848
Communist party membership (no = ref.)					−0.0055	0.0907	−0.0118	0.0907
**Built environments**								
Population density			−0.3239^***^	0.0958	−0.3119^***^	0.0957	−0.2047^*^	0.9305
Land use mix			5.2960^***^	0.8247	5.1335^***^	0.8213	5.1482^***^	0.9305
Accessibility of open space			0.0004^**^	0.0001	0.0004^**^	0.0001	0.0003^*^	0.0002
Accessibility of kindergarten			0.0861^***^	0.0253	0.0805^***^	0.0252	0.0557^*^	0.0288
Accessibility of public transport			−0.2733^***^	0.0937	−0.2578^***^	0.0936	−0.3146^***^	0.1055
Female × population density							−0.1628^*^	0.0976
Female × accessibility of kindergarten							0.0412^*^	0.0246
Intercept	−0.0009	0.1622	−0.0012	0.0690	−0.1552	0.2443	−0.1420	0.2431
Between-group variance	0.3029	0.1289	0.0443	0.0233	0.0436	0.0231	0.0407	0.0220
Within-group variance	0.6521	0.3768	0.6521^***^	0.0377	0.6204^***^	0.0359	0.6144	0.0355
ICC (%)	31.72%		6.37%		6.57%		6.21%	

^*^p < 0.1, ^**^p < 0.05, ^***^p < 0.01. ICC = between-group variance/(between-group variance + within-group variance).

#### 4.2.1 The impact of the built environments on SWB

Model 1 is the model that adds the built environment variables into the empty model. In this model, the between-group variance decreases to 0.0443, and the ICC drops to 6.37%, indicating that the selected built environment variables can effectively explain the heterogeneities of SWB at the community level among older adults. In other words, the differences of SWB at the community level are largely due to variations in the built environment.

Specifically, population density is significantly negatively correlated with the SWB of older adults (β = −0.3239, *p* < 0.01). Existing studies indicate that SWB is inverted *U*-shaped in population density (50, 51). That is, SWB level tends to increase with population density, reaches the peak, and then decreases while the population density increase. Studies based on Western cases often find a positive correlation between population density and SWB (56–58), for the reason that the population densities there are generally low, reflecting the first half of the inverted “*U*”. In our case, the population densities of the communities are all very high. The results here actually reflect another half of the inverted “*U*”-the negative relationship between population density and SWB. Land use mix is significantly positively correlated with SWB (β = −0.3239, *P* < 0.01), consistent with the literature (59, 60). The reason could be that mixed land use can provide convenient living conditions for older adults and the proximity of destinations promotes social interaction, thereby improving their psychological health.

Regarding the accessibility of the open space, the result shows that the further away from open space, the higher the subjective wellbeing of older adults (β = 0.0004, *P* < 0.05), which contradicts with existing research (61, 62). On the one hand, open spaces provide good conditions for exercise and recreation, facilitating physical and social activity involvements, thus increasing SWB of older adults. On the other hand, in China older adult people, especially women, often gather in open space to dance with loud background music. Being closer means being more susceptible to the noisy and crowded living conditions, potentially lowering their SWB. The two pathways have contradicted effects on SWB and the negative influence of proximity to open space dominate the overall results.

Accessibility to kindergartens is found to be positively correlated with SWB (β = 0.0861, *P* < 0.01), perhaps because having more kindergartens within walking distance makes it more convenient for older adults to drop off and pick up children, thus positively affecting their level of SWB. Regarding accessibility to metro stations, the more subway stations around the community, the lower the SWB level of older adults. Like population density, previous research has shown that accessibility to subway stations could have both positive and negative impacts on subjective wellbeing (56, 63). In this paper, the negative effect may be because that communities closer to subway stations typically have higher building densities, more shops, and larger traffic flows, and therefore lead to a decline in SWB of older adults.

In order to examine whether built environments have different impacts on the SWB of older adult men and women, we conducted regression analyses separately for older adult females and males (model results are not presented here due to limited space). Results indicate that community-level variables can explain 28.50% of the total variance in SWB for older adult men and 33.68% for older adult women. All built environment variables had significant impacts (*P* < 0.01) on the SWB of older adult women, while population density had no significant effects on men's SWB. Compared to older adult men, there are greater differences in SWB at the community level and the built environments exert more prominent impacts on SWB of older adult women.

To further explore how specific attribute of built environments impact SWB of older adult men and women, we established interactions between the built environment variables and gender to investigate the moderating role, as shown in Model 3. Results show that there are gender differences in the impacts of the built environments on the wellbeing of older adults: the coefficient for the interaction between women and population density is negative and significant at the 10% level, indicating that as population density increases the SWB of older adult women reduces more than that of men. Moreover, the interaction between women and accessibility to kindergartens is positive and significant at the 10% level, suggesting that accessibility to these facilities has a more pronounced effect on enhancing the wellbeing of older adult women compared to men.

#### 4.2.2 The impact of household structure on SWB

Model 2 incorporates seven types of household structure at the individual level and controls for socio-demographic variables. In the model, living with daughter-in-law is taken as reference group. The levels of SWB among older people in different household structures rank from high to low as follows: living with daughter-in-law, others, living with spouse, living with daughter, living with daughter-in-law and grandchildren (β = −0.2826, *P* < 0.1), living alone (β = −0.3055, *P* < 0.1), living with daughter and grandchildren (β = −0.3279, *P* < 0.05). Pairwise compared, older people living with daughter-in-law have high SWB than those living with daughter-in-law and grandchildren, and older people living with daughter are more satisfied with their life than those living with daughter and grandchildren. It seems that the presents of the grandchildren tend to reduce the SWB level of older adults. This might be related to more household tasks allocated to older adults when there are grandchildren in the family. Living with daughter or living with daughter-in-law also influences old people's SWB. When pairwise compared, older people living with daughter-in-law have high SWB than those living with daughter, and older people living with daughter-in-law and grandchildren are more satisfied with their life than those living with daughter and grandchildren. China is a country with a robust tradition of extended family and patriarchal and patrilocal living arrangements. This tradition is ascribed to Confucian doctrines that emphasize children's filial obligations to their parents, particularly those of sons. To live with sons are considered successful aging and therefore positively associated with wellbeing. Obviously, household structure is an important factor influencing older adults' SWB.

We also conducted regression analyses separately for older adult females and males to investigate whether household structures have different impacts on the SWB of older adult men and women (model results are not presented here due to limited space). Results show that the presences of grandchildren seem reduced the SWB of the older adult men more substantially than that of the older adult women. The reason could be that old men have to share the additional housework stemming from the existence of the grandchildren.

## 5 Discussion

The above analyses show that there are indeed significant gender differences in SWB of older adults: older adult women have higher levels of SWB than older adult men. Built environment and household structure are found to be important factors affecting SWB of older adults, and their influences are also gendered. In the following, we proposed three mechanisms that can explain these empirical results.

### 5.1 Structural mechanisms

The structural mechanism refers to discrepancies between old men and women in resource acquisition, education attainment, opportunities of employment and social-economic status in the society. Existing research indicates that SWB of older adults varies with the degree of gender inequality and people's attitudes toward gender equality in the society (20): the more positive the attitude toward gender equality and the smaller the gender inequality, the smaller the gender difference in subjective wellbeing (20, 64). The old women of our respondents are of the cohorts who have spent most of their lives in a relatively disadvantaged society. Compared with old men, they generally have lower expectations of SWB (17). However, in recent decades, women's social and economic conditions have greatly improved. Gender inequality in China has significantly decreased. The advantages of men in resource acquisition, employment opportunities, and social status have sharply declined. The above changes may be the reason why the SWB of older adult women has significantly improved and exceeded that of older adult men (30). Another structural change in later life is that the dominant person in the family often reverses from men to women. While men traditionally hold dominance, their economic advantage diminishes as they enter retirement. Moreover, older adult men become increasingly reliant on older adult female caregivers for their daily needs, which in turn elevates the status of women within the household. The changing roles in the family narrows the gender-related gaps to some extent and enhance the SWB of older adult women.

### 5.2 Socio-cultural mechanisms

The socio-cultural mechanism pertains to the manner in which gender roles and norms, as shaped by social and cultural forces, give rise to disparities in subjective wellbeing among older adults. Gender roles refer to the social and behavioral norms that are widely considered to be socially appropriate for individuals of a specific sex within a specific culture (65). These roles dictate how men and women should behave, dress, speak, interact, and fulfill their duties in society based on their gender. There are at least two pathways through which gender roles influence the subjective wellbeing of older adults. Firstly, gender roles could influence the division of household works between men and women and consequently impact their subject wellbeing. In the Chinese traditional norms, men tend to provide economic support, while women bear more responsibility for caring for the family and raising children (9, 38, 66). As shown in Table 5, older adult women tend to share considerably more household chores compared with older adult men in the same household type. Meanwhile, the household burdens carried by older adult women are not changed along with household types as dramatically as those carried by the older adult men. It can be understood that older adult women, accustomed to this labor burden, are not significantly affected by changes in housework due to changes in household structure. Nevertheless, changes in the type and intensity of housework tend to significantly affect the happiness of older adult men. This explains the model result those older adult men living with a daughter-in-law and grandchildren have a significantly lower level of happiness compared to those living with daughter-in-law, while there is no significant change for older adult women.

**Table 5:** The proportion of older adults participating in housework in different household structures.

**Housework**	**Gender**	**Single**	**Couple**	**Daughter**	**Daughter-in-law**	**Daughter and grandchildren**	**Daughter-in-law and grandchildren**	**Others**	**Total**
Sharing housework	Male	53.8%^*^	84.4%^*^	75.0%^*^	86.7%^*^	81.0%^*^	88.9%^*^	74.1%^*^	81.8%^*^
	Female	94.4%	95.0%	89.7%	92.3%	100.0%	96.0%	92.6%	94.6%
Laundering, cleaning, cooking	Male	85.7%	86.4%	75.0%	84.6%	94.1%	81.3%	95.0%	85.9%
	Female	100.0%	98.9%	94.3%	100.0%	94.9%	91.7%	98.0%	96.6%
Taking care of children	Male	0.0%^***^	18.5%^***^	41.7%^***^	53.8%^***^	76.5%^***^	70.8%^***^	50.0%^***^	42.4%^***^
	Female	5.9%^***^	20.0%^***^	31.4%^***^	37.5%^***^	84.6%^***^	76.4%^***^	38.0%^***^	42.4%^***^
Sending and picking up children	Male	0.0%^***^	12.3%^***^	33.3%^***^	38.5%^***^	47.1%^***^	41.7%^***^	20.0%^***^	25.8%^***^
	Female	2.9%^***^	14.7%^***^	20.0%^***^	16.7%^***^	53.8%^***^	45.8%^***^	22.0%^***^	26.1%^***^
Daily shopping	Male	42.9%	60.5%	58.3%	61.5%	76.5%	54.2%	50.0%	58.6%
	Female	70.6%^*^	74.7%^*^	68.6%^*^	50.0%^*^	87.2%^*^	84.7%^*^	72.0%^*^	75.1%^*^

^*^p < 0.1, ^***^p < 0.01.

Moreover, gender differences in the division of household tasks lead to differing demands for the built environments and facilities. Since women mainly take on the responsibility of caring for the family and raising children, they rely more on the surrounding built environment (67) and consequently the built environment has more significant impacts on the subjective wellbeing of older adult women. This also explains why population density and accessibility to kindergartens demonstrates a greater impact on their wellbeing in the model.

Secondly, the socio-cultural norms could change the level of wellbeing of older adults by influencing their preference and attitudes toward different patterns of older adult care and living arrangements in later life. Chinese society emphasizes cohabitation and mutual support between generations (5, 42), and living with adult children, especially sons, is often seen as a necessary prerequisite for older adult people to obtain intergenerational support and ensure happiness in later life. Hence, most older adult people tend to live with adult children, especially sons (68), and believe that cohabiting with children can yield a higher social evaluation and is considered a “successful” pattern to aging (43). This may explain why the subjective wellbeing of older adults living with daughter-in-law are higher than those living with daughter, and those living with daughter-in-law and grandchildren are much happier than those living with daughter and grandchildren when pairwise compared. Overall, socio-cultural mechanism, especially the concept of filial piety and the division of household tasks, are important pathways through which household structure and the built environment have significant gender differences in their impacts on the wellbeing of older adults.

### 5.3 Physiological mechanisms

Previous studies suggest that differences in the physiological factors such as hormonal and genetic structures between men and women may directly lead to variations in subjective wellbeing between the sexes (69, 70). Due to these differences, women tend to pay more attention to self-expression, intimacy and support compared to men (71). They also have the tendency toward altruism and find that closeness to family and helping others have a more significant positive impact on their subjective wellbeing (40, 41). Therefore, there is a significant increase in levels of happiness for older adults women living with daughters-in-law as compared to those living with spouses. The positive effects of fulfilling emotional needs even surpass the negative impacts caused by an increased burden of household chores. On the other hand, as they age, individuals experience a decline in physical and perceptual abilities, and their daily activity spaces gradually shrink (55, 72). Comparison to older adult men, older adult women have an even smaller ranges of activities and are more dependent on the built environment at the community scale (72, 73), making them more susceptible to its influences (73). The model results of this paper suggest that the built environment at the community level has a more pronounced impact on the wellbeing of older adult women, which also echoes the findings of existing research (55, 73).

## 6 Conclusions

Subjective wellbeing serves as a vital metric for evaluating the psychological health of senior citizens. Notable disparities in life expectancy and subject wellbeing between men and women highlight the necessity for gender-sensitive public policies that aim to uplift the wellbeing and health of the aging population. Based on a survey of the quality of life of older adults in Nanjing, this study finds significant gender differences in the subjective wellbeing of older adults. Built environment and household structures are found to exert markedly different influences on the happiness of older men and women. Structural mechanisms, socio-cultural mechanisms, and physiological mechanisms are the three main factors contributing to these gender differences. China's unique built environment, social and cultural background profoundly affect the cognition, preferences, and behavior patterns of older adults, thus resulting in their differences in perceptions of wellbeing and the underlying mechanisms from those in the West. This paper reveals the patterns and mechanisms of the gender differences in senior's wellbeing in the Chinese context, and also, to some extent, enriches the empirical research and theories related to subjective wellbeing.

The results can provide references in formulating policies to create age-friendly community and enhance the health and welfare of older adults for both sexes. Firstly, the high-density built environment in China is quite different from that in West, and the excessive population density has a negative impact on the happiness of older adults, especially on older women. Therefore, policies should not only focus on the provisions of various facilities but also on using measures such as constructing green belts and regulating behavioral norms for some activities especially Square Dance to reduce negative impacts of high density. Moreover, given the significant gender differences in the impacts of the built environment on older adults' SWB, it is essential to consider these differences in the patterns of daily behavior and the consequent facility needs between men and women. Environmental interventions, such as increasing land-use diversity and accessibility of related facilities should be implemented to reduce the burden of domestic labor and to facilitate independent life for older adults, especially older women.

It is important to note that the household structure we studied is the reflection of domestic labor, family comfort and other factors affecting the older adults' SWB. However, living apart does not necessarily prevent children from providing financial and emotional support to older adults. In addition, regarding the variables of the built environment, our study solely focuses on the impact of the objective built environment on SWB and ignores the subjective built environment. In the future more sophisticated survey will be conducted to explore the impacts of both the objective and subjective built environments on senior's subjective wellbeing.

## Data Availability

The raw data supporting the conclusions of this article will be made available by the authors, without undue reservation.
